# Fetal sex and maternal postpartum depressive symptoms: findings from two prospective pregnancy cohorts

**DOI:** 10.1186/s13293-020-00348-x

**Published:** 2021-01-06

**Authors:** Whitney Cowell, Elena Colicino, Talia Askowitz, Farida Nentin, Rosalind J. Wright

**Affiliations:** 1 Department of Environmental Medicine and Public Health, Icahn School of Medicine at Mount Sinai, New York, NY USA; 2 Department of Pediatrics, Kravis Children’s Hospital, Icahn School of Medicine at Mount Sinai, New York, NY USA; 3 Department of Obstetrics and Gynecology, Icahn School of Medicine at Mount Sinai, New York, NY USA

**Keywords:** Depression, Fetus, Postnatal, Postpartum, Pregnancy, Sex

## Abstract

**Background:**

Fetal sex is known to modify the course and complications of pregnancy, with recent evidence of sex-differential fetal influences on the maternal immune and endocrine systems. In turn, heightened inflammation and surges in reproductive hormone levels associated with pregnancy and parturition have been linked with the development of perinatal depression. Here, we examined whether there is an association between fetal sex and maternal depression assessed during the prenatal and postnatal periods.

**Methods:**

The study included two multi-ethnic, prospective pregnancy cohorts that enrolled women from prenatal clinics in the Northeastern United States between 2001 and 2018. Maternal depressive symptoms were measured during the prenatal and postnatal periods using the Edinburgh Postpartum Depression Scale (EPDS), and newborn sex was reported by the mother following delivery. We used logistic regression to examine associations between fetal sex and maternal depressive symptoms (EPDS > 10) during the prenatal period only, postnatal period only, or both periods versus no depressive symptoms during either period. We considered both unadjusted models and models adjusted for a core set of sociodemographic and lifestyle variables.

**Results:**

In adjusted models using PRISM data (*N* = 528), women pregnant with a male versus female fetus had significantly greater odds of depressive symptoms during the postnatal period compared to women without depressive symptoms during either period (odds ratio [OR] = 5.24, 95% confidence interval [CI] = 1.93, 14.21). The direction of results was consistent in the ACCESS cohort, although the findings did not reach statistical significance (OR = 2.05, 95% CI = 0.86, 4.93). Significant associations were not observed in either cohort among women with prenatal symptoms only or women with prenatal and postnatal symptoms.

**Conclusions:**

Male fetal sex was associated with the onset of depressive symptoms during the postnatal period.

**Supplementary Information:**

The online version contains supplementary material available at 10.1186/s13293-020-00348-x.

## Background

Perinatal depression, defined here as the onset of major depressive disorder during or following pregnancy, is one of the most prevalent pregnancy-related morbidities. Research studies estimate approximately 10–19% of women develop symptoms within 3 months of delivery, although prevalence estimates vary depending on study design and definitions used [1, 2]. In addition to affecting the mother’s emotional health and well-being, perinatal depression precipitates other adverse physical, psychological, social, and economic outcomes for both the mother and child with potentially long-lasting consequences [3]. For example, maternal depression experienced during pregnancy has been associated with obstetric complications [4], restricted fetal growth [5], altered neurodevelopmental programming [6], and impaired infant bonding [3].

During pregnancy, the maternal system undergoes a series of carefully orchestrated adaptations necessary to sustain fetal growth and development. Increasingly, signals from the placenta and fetus are thought to play key roles driving these changes and may contribute to pregnancy-related disease risk [7]. For example, paternally derived fetal antigens elicit maternal immune system activity [8], with research suggesting tolerance of the semi-allogenic fetus is in part coordinated through the release of placental extracellular vesicles into the maternal and fetal circulations [9]. The microRNAs and other signals carried by these vesicles interact with distal recipient cells to modify maternal metabolism and physiology and have been associated with a range of reproductive- and obstetric-related disorders [10, 11].

Research focused on perinatal depression specifically provides evidence that women who exhibit symptoms show an exaggerated inflammatory response around the time of delivery [12–14] and have altered levels of placental enzymes that regulate maternal-fetal hormone and neurotransmitter transfer [6]. Clinical research has also shown that fluctuations in reproductive and stress hormones, which occur gradually across the course of gestation and dramatically during parturition, contribute to system dysregulation and the onset of depressive symptoms in susceptible women [15]. For example, a double-blind pregnancy-simulation study (*n* = 16) demonstrated that depression symptoms could be induced via administration and withdrawal of synthetic estrogen and progesterone among women with a history of perinatal depression [16].

Expanding research supports that fetal and placental influences on the maternal system may be sex-differential [17]. For example, male fetal sex has been linked to chronic placental inflammation [18] and higher levels of pro-inflammatory cytokines in the maternal circulation [19]. Similar research has identified differential shifts in maternal reproductive hormone levels by fetal sex [20–23]. Given that maternal immune activation and fluctuations in reproductive hormones during pregnancy and parturition are implicated in the pathophysiology of perinatal depression [24–27], coupled with research supporting fetal sex-specific influences on maternal immune and endocrine systems during pregnancy, we hypothesized that maternal perinatal depressive symptoms would vary by newborn sex, with the prevalence of elevated symptoms greater among male pregnancies. We further based our hypothesis on recent evidence from a retrospective survey that found the odds of postnatal depression were significantly greater among women carrying a male fetus [28]. Given that risk factors related to the onset of perinatal depression, such as shifts in hormone levels [26, 27] and immune activity [29], may vary across the course of gestation and into the postpartum period, we considered the importance of timing by examining depressive symptomology measured during both the prenatal and postnatal periods.

## Methods

### Study cohorts

Analyses included women enrolled in one of two pregnancy cohorts based in Boston, USA, and New York City, USA, with similarly derived information on fetal sex and maternal depressive symptoms.

#### *Asthma Coalition on Community Environment and Social Stress* (ACCESS)

English- or Spanish-speaking women (≥ 18 years) with a singleton pregnancy were enrolled from Brigham and Women’s Hospital (BWH), Boston Medical Center (BMC), and affiliated prenatal clinics from 2001 to 2010. *N* = 961 mothers delivered a live born infant and remained eligible for study follow-up. Procedures were approved by the human studies committees at BWH and BMC; written informed consent was obtained in the mother’s preferred language.

#### *Programming of Intergenerational Stress Mechanisms* (PRISM)

Pregnant English- or Spanish-speaking women (≥ 18 years) with a singleton pregnancy were recruited from prenatal clinics at the East Boston Neighborhood Health Center, the BWH, and the Beth Israel Deaconess Medical Center (BIDMC) in Boston and from the Mount Sinai Hospital in New York City. Exclusion criteria included HIV-positive status or drinking > 7 alcoholic drinks per week prior to pregnancy recognition or any alcohol after pregnancy recognition. Recruitment began in 2011 and is ongoing; the present study considered *N* = 989 eligible women with a live born infant enrolled through December 2019. Procedures were approved by the institutional review boards at BWH and the Icahn School of Medicine at Mount Sinai; BIDMC relied on BWH for review and oversight of the protocol. Written informed consent was obtained in the mother’s preferred language.

In ACCESS, women excluded due to missing depression assessments (*n* = 624, 64%) were more likely to be Black/Black-Hispanic (34% vs. 28%), and less likely to be Hispanic, non-Black (49% vs. 59%) or to have breastfed for more than 6 months (47% vs. 64%) (Supplemental Table 1). Similarly, in PRISM, excluded women (*n* = 459, 46%) were more likely to be Black/Black-Hispanic (51% vs. 38%), and less likely to be White, non-Hispanic (12% vs. 20%), to be over age 35 years (15% vs. 20%), or to have breastfed for more than 6 months (26% vs. 57%) (Supplemental Table 2). The differences in age and breastfeeding duration between excluded and included women likely reflect differences in race/ethnicity, as Black/Black-Hispanic women in both cohorts were younger and less likely to breastfeed for more than 6 months.

### Maternal perinatal depression symptoms

**Fig. 1:**
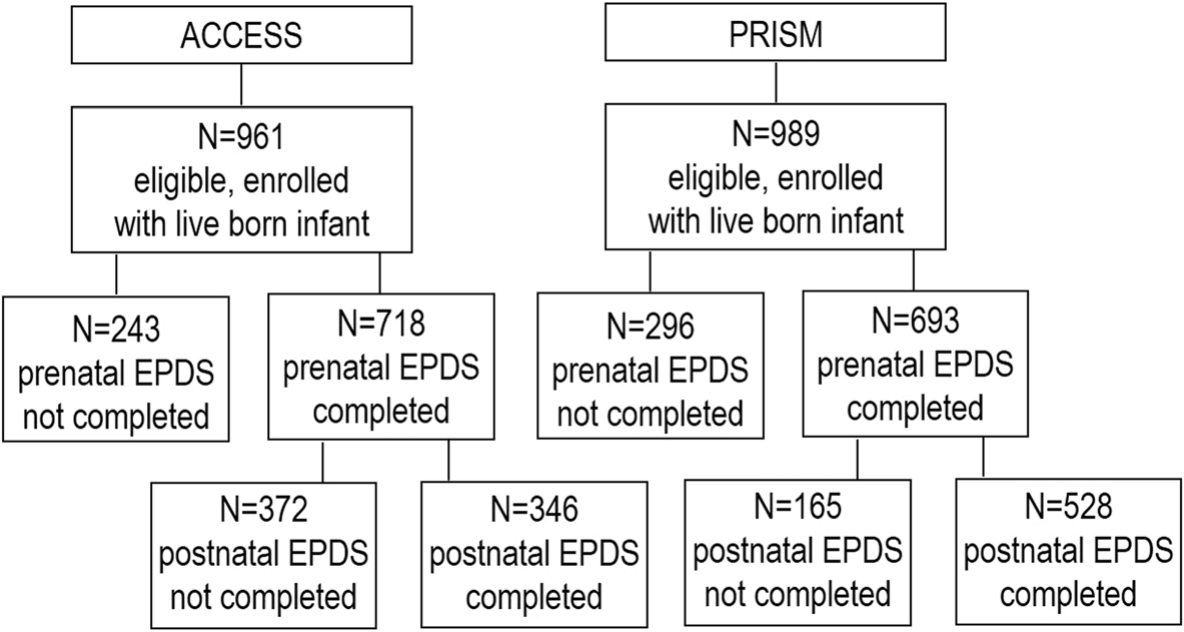
Diagram of analytic sample selection from the PRISM and ACCESS pregnancy cohorts. *Abbreviations*: ACCESS, Asthma Coalition on Community Environment and Social Stress; EPDS, Edinburgh Postnatal Depression Scale; PRISM, PRogramming of Intergenerational Stress Mechanisms

We measured maternal depression symptoms in-person or by telephone during the prenatal (mean ± SD, ACCESS 27.3 ± 8.2 weeks gestation, PRISM 29.0 ± 8.0 weeks gestation) and postnatal (ACCESS 5.0 ± 1.6 months postpartum, PRISM 6.6 ± 0.8 months postpartum) periods using the Edinburgh Postpartum Depression Scale (EPDS). The EPDS was developed as a screening assessment for identifying women with self-reported perinatal depressive symptoms [30]. The validated tool is available in English and Spanish and has sensitivity and specificity of 95% and 93% compared to the Diagnostic and Statistical Manual of Mental Disorders, Third Edition criteria, respectively [30–32]. Women rated each of 10 questions on a 4-point scale (0–3) reflecting how often she experienced various symptoms associated with depression during the previous 7 days. Consistent with prior studies, we defined elevated depressive symptomology as an EPDS score > 10 [33]. Of the 961 women enrolled in ACCESS, 718 (75%) completed the EPDS during pregnancy and 346 (36%) additionally completed the EPDS during the postnatal period (Fig. 1). Of the 987 eligible women enrolled in PRISM, 693 (70%) completed the EPDS during pregnancy and 528 (53%) additionally completed the EPDS during the postnatal period (Fig. 1). Prenatal EPDS scores did not significantly differ between women with versus without a postnatal measure in either cohort (Wilcoxon rank sum: ACCESS: *Z* = 0.58, *p* = 0.56; PRISM: *Z* = 1.13, *p* = 0.26). Only women with complete EPDS measures at both periods were included in the study.

Mothers were additionally asked a series of questions about depressive feelings, depression diagnosis, and the use of prescription medication for depression before and during pregnancy (PRISM only). Specifically, mothers were asked: “Before this pregnancy, was there ever a period of time when you were feeling depressed or down or when you lost interest in pleasurable activities most of the day, nearly every day, for at least 2 weeks?”, “Before this pregnancy, did you ever see a health care professional who said that you were depressed?”, and “During your pregnancy, did you take prescription medication for depression or to help you sleep?”

### Child sex

Child sex was reported by the mother at a study visit conducted approximately 1 month following delivery. In the PRISM cohort, self-reported newborn sex was confirmed by a review of the baby’s medical record.

### Covariates

Data on sociodemographic characteristics (race/ethnicity, age, education, relationship status) and lifestyle factors (parity, pre-pregnancy body mass index [BMI], smoking during pregnancy) were collected using questionnaires administered in-person by trained study staff during pregnancy. Information on breastfeeding duration was assessed by in-person or telephone questionnaires administered through age 6 months. In PRISM, information on admittance to the Neonatal Intensive Care Unit (NICU) and gestational age at birth was abstracted from the newborn’s medical record. Gestational age was based on best obstetrical estimate derived from routine prenatal ultrasounds; if no obstetrical estimate was available, gestational age was calculated from the date of birth and maternal reported last menstrual period.

### Statistical analyses

We calculated descriptive statistics for all sociodemographic and lifestyle characteristics considered. We examined the distribution of maternal EPDS scores using histograms and boxplots and calculated Spearman correlations between prenatal and postnatal scores. We created a four-level categorical variable that indicated the presence of depressive symptoms (defined as an EPDS score > 10) during the prenatal and/or postnatal period as follows: (1) no depressive symptoms during either period, (2) depressive symptoms during the prenatal period only, (3) depressive symptoms during the postnatal period only, and (4) depressive symptoms during both periods. In each cohort considered separately, we used logistic regression to examine the odds of maternal depressive symptoms during the prenatal period only, postnatal period only, or both periods versus no depressive symptoms during either period among women carrying a male versus female fetus. We used the Firth penalized maximum likelihood estimation method to reduce potential bias introduced by sparse cases across the four levels of prenatal versus postnatal depressive symptoms [34]. Given that there are few known determinants of fetal sex, the relationship between fetal sex and maternal depressive symptoms is unlikely susceptible to confounding by shared antecedents of the exposure and outcome. Here, we examined both unadjusted models, as well as models adjusted for a core set of sociodemographic variables (maternal race/ethnicity [White, non-Hispanic/other vs. Black/Black-Hispanic vs. Hispanic, non-Black], age [< 35 vs. ≥ 35 years], education [< high school vs. ≥ high school]) and lifestyle or health-related precision variables that have previously been associated with depression, including relationship status (married or living with partner vs. others), parity (nulliparous vs. multiparous), pre-pregnancy BMI (continuous in kg/m^2^), cigarette smoking during pregnancy (any vs. none), and breastfeeding duration (≤ 6 months vs. > 6 months). For regression models, women who self-reported their race/ethnicity as “other” (approximately 5%) were collapsed with white women due to small numbers across levels of the depression variable. In adjusted models, we used multiple imputation with chained equations to impute values for missing covariates (ACCESS: *n* = 47, 14%; PRISM: *n* = 26, 5%).

To understand potential misclassification related to our use of the EPDS in these samples, we used separate multinomial logistic regression models to examine maternal self-reported feelings of depression before pregnancy, diagnosis with depression (ever) before pregnancy, or use of depression medication during pregnancy (PRISM only) in relation to the 4-level prenatal/postnatal EPDS variable.

Evidence suggests that boys may be more susceptible to neonatal complications, which in turn may lead to increased risk for maternal depression. To evaluate this possibility, we examined bivariate associations between neonatal complications, defined as premature birth (< 37 weeks gestation) or admittance to the NICU (yes/no) and maternal EPDS scores in the PRISM cohort. All statistical analyses were performed using SAS v9.4.

## Results

Across cohorts, the majority of women were minorities (ACCESS = 91%, PRISM = 80%) and approximately one-quarter to one-third had less than a high school education (ACCESS = 35%, PRISM = 23%). Table 1 provides additional sociodemographic and lifestyle characteristics of each cohort stratified by fetal sex. Prenatal and postnatal EPDS scores were log-normally distributed and moderately correlated (ACCESS: *ρ* = 0.30, *p* < 0.0001; PRISM: *ρ* = 0.39, *p* < 0.0001). More women presented with elevated depressive symptoms during the prenatal (ACCESS 26%, PRISM 22%) versus the postnatal (ACCESS 17%, PRISM 13%) period. Additionally, the prevalence of depressive symptoms varied by maternal race/ethnicity for both the ACCESS and PRISM cohorts, with Black and Hispanic mothers typically having higher scores (Supplemental Table 3). In both cohorts, women with a high school or more education were more likely to have a boy, and in the PRISM cohort, older women at the time of delivery were less likely to have a boy (Table 1). No other sociodemographic or lifestyle characteristics considered significantly varied by fetal sex.

**Table 1:** Characteristics (*N* [%]) of ACCESS and PRISM mother-child pairs included in analyses stratified by fetal sex

	ACCESS^a^				PRISM^b^			
	All (***N*** = 346)	Male (***N*** = 188)	Female (***N*** = 158)	***p*** value^**c**^	All (***N*** = 528)	Male (***N*** = 273)	Female (***N*** = 255)	***p*** value^**c**^
Maternal race/ethnicity				0.297				0.473
White, non-Hispanic	30 (8.8)	14 (7.8)	16 (10.3)		102 (19.5)	56 (20.6)	46 (18.3)	
Black/Hispanic-Black	95 (27.9)	59 (31.9)	36 (23.1)		200 (38.2)	102 (37.5)	98 (39.0)	
Hispanic, non-Black	200 (58.7)	103 (55.7)	97 (62.2)		193 (36.9)	96 (35.3)	97 (38.7)	
Others	16 (4.7)	9 (3.9)	7 (4.5)		28 (5.4)	18 (6.6)	10 (4.0)	
High school or more	218 (65.1)	128 (69.6)	90 (59.6)	0.057	400 (77.2)	217 (81.0)	183 (73.2)	0.035
Advanced maternal age (> 35 years)	40 (11.6)	23 (12.3)	17 (10.8)	0.672	103 (19.5)	44 (16.1)	59 (23.1)	0.042
Pre-pregnancy BMI > 24 kg/m^2^	217 (69.8)	116 (69.1)	101 (70.6)	0.762	278 (53.2)	142 (52.6)	136 (53.8)	0.790
Smoking during pregnancy	41 (11.9)	22 (11.7)	19 (12.1)	0.909	64 (12.1)	34 (12.5)	30 (11.8)	0.808
Married/living with partner	210 (61.8)	116 (62.3)	94 (61.0)	0.802	362 (69.4)	186 (69.1)	176 (69.6)	0.917
Nulliparous	146 (43.3)	80 (43.7)	66 (42.9)	0.874	160 (30.5)	90 (33.1)	70 (27.7)	0.178
Preterm	37 (10.8)	22 (11.8)	15 (9.5)	0.486	46 (8.7)	27 (9.9)	19 (7.5)	0.321
Breastfeed > 6 months	154 (63.6)	83 (63.9)	71 (63.4)	0.942	293 (56.7)	151 (56.8)	142 (56.6)	0.965
Perinatal depression^c^				0.029				0.015
No symptoms	228 (65.9)	122 (64.9)	106 (67.1)		390 (73.9)	194 (71.0)	196 (76.9)	
Prenatal only	58 (16.8)	34 (18.1)	24 (15.2)		71 (13.5)	38 (13.9)	33 (12.9)	
Postnatal only	27 (7.8)	20 (10.6)	7 (4.4)		24 (4.6)	20 (7.3)	4 (1.6)	
Prenatal and postnatal	33 (9.5)	12 (6.4)	21 (13.3)		43 (8.1)	21 (7.7)	22 (8.6)	

*Abbreviations*: *ACCESS* Asthma Coalition on Community Environment and Social Stress, *BMI* body mass index, *EPDS* Edinburgh Postpartum Depression Scale, *PRISM* PRogramming of Intergenerational Stress Mechanisms

^a^ACCESS (*n* missing): race/ethnicity (5), age (2), education (11), BMI (35), smoking (1), relationship status (6), parity (9), and breastfeeding (104)

^b^PRISM (*n* missing): race/ethnicity (5), education (10), BMI (5), relationship status (6), parity (3), and breastfeeding (11)

^c^*p* values are from chi-square tests of independence

^d^Defined as an EPDS score > 10

In both cohorts, women who developed postnatal depressive symptoms, but who did not exhibit symptoms during pregnancy, were significantly more likely to carry a male fetus (Table 1). In adjusted logistic regression models, the odds of postnatal depressive symptoms increased after the birth of male infants by 105% in ACCESS and 424% in PRISM; however, the association was not statistically significant in ACCESS (Table 2). Results from unadjusted models were similar in direction and magnitude.

**Table 2:** Odds of perinatal depressive symptoms (defined as an Edinburgh Postpartum Depression Scale score > 10) among women pregnant with a male versus female

	Unadjusted	Adjusted^**a**^
	OR (95% CI)	OR (95% CI)
**ACCESS**		
No symptoms (*n* = 228, 66%)	1.00 (reference)	1.00 (reference)
Prenatal only (*n* = 58, 17%)	1.22 (0.69, 2.19)	1.19 (0.65, 2.16)
Postnatal only (*n* = 27, 8%)	2.38 (0.99, 5.73)	2.05 (0.86, 4.93)
Prenatal and postnatal (*n* = 33, 9%)	0.51 (0.24, 1.07)	0.52 (0.24, 1.10)
**PRISM**		
No symptoms (*n* = 390, 74%)	1.00 (reference)	1.00 (reference)
Prenatal only (*n* = 71, 13%)	1.16 (0.70, 1.92)	1.13 (0.67, 1.90)
Postnatal only (*n* = 24, 5%)	4.60 (1.62, 13.05)	5.24 (1.93, 14.21)
Prenatal and postnatal (*n* = 43, 8%)	0.97 (0.52, 1.80)	0.91 (0.48, 1.74)

^a^Adjusted for race/ethnicity, age, education, parity, relationship status, smoking during pregnancy, body mass index, and breastfeeding duration

As expected, women who reported feeling depressed before pregnancy (ACCESS: *n* = 132, 38%; PRISM: *n* = 215, 41%) or who had ever been diagnosed with depression by a clinician (ACCESS: *n* = 77, 22%; PRISM: *n* = 128, 24%) were significantly more likely to exhibit depressive symptoms during the prenatal and/or postnatal periods, as determined by the EPDS, with the highest odds among women exhibiting symptoms during both periods (Supplemental Table 4). In PRISM, women who took prescription medication for depression during pregnancy (*n* = 35, 7%) were also more likely to exhibit prenatal and postnatal symptoms or postnatal symptoms only (Supplemental Table 4). These findings support our use of the EPDS as a tool for assessing maternal depression in these samples. In the PRISM cohort, *n* = 104 (20%) babies were born premature and/or admitted to the NICU; we did not detect an association between fetal sex and these outcomes (odds ratio (OR) = 1.11, 95% confidence interval (CI) 0.72, 1.71).

## Discussion

In this prospective study, we found that women giving birth to a male versus a female infant were more likely to develop depressive symptoms during the postnatal period. These results are consistent with a recent, retrospective study (*n* = 296 women, 651 births) conducted in the UK that found the odds of postpartum depression (PPD) were 71–79% higher among women carrying a male versus female fetus, after controlling for other risk factors and individual-level characteristics [28]. Similarly, a cross-sectional study of 181 women based in France found postnatal depression was significantly associated with having a male infant [35] and a study of 2267 women in Sweden found male infant sex was associated with an increased odds of experiencing postpartum depressive symptoms within 5 days of delivery, but not at 6 weeks or 6 months postpartum [36]. In the present study, we found that women carrying a male fetus also had a higher odds of prenatal depressive symptoms, although the magnitude of this association was small and did not reach statistical significance. The stronger findings observed with depressive symptoms during the postnatal, versus prenatal, period could suggest that fetal-sex-linked events during late pregnancy or parturition may specifically contribute to PPD susceptibility. Notably, the majority of prior research has not been designed to examine temporal variation in associations across the pregnancy and postpartum periods.

Several biological mechanisms could underlie our observed findings, including sex-differential shifts in maternal reproductive and other hormones or immune activity. The Reproductive Hormone Model of PPD, which is supported by multiple lines of research, including animal models, clinical trials, and biomarker-based observational studies, purports that PPD arises among a sensitive phenotype of women following hormonal fluctuations in the brain that occur postpartum and during other reproductive phases (i.e., premenstrual and peri-menopausal periods) [26, 27]. For example, following parturition, estradiol, which modulates the serotonergic system, and progesterone levels rapidly decrease [37]. Research conducted in rats has shown experimental induction of a hormone withdrawal state that mimics that of the postpartum period results in displays of depressive behavior, despair, and anhedonia [38–41]. Corresponding research in humans has shown pharmacologically neutralizing estrogen withdrawal following delivery reduces the onset of depressive symptoms among women with a history of PPD [42]. Notably, recent research has demonstrated that fetal sex modifies the association between reproductive hormone profiles across pregnancy and maternal behavior during the postpartum period [43], providing evidence that the fetus may influence maternal neurobiology with lasting effects. Other research has reported sex-differential levels of reproductive hormones during pregnancy in maternal serum [22, 44–46] and amniotic fluid [47]. For example, several studies have shown higher serum estrogen levels in female-bearing pregnancies [22, 44, 45]. It is plausible that the lower pregnancy estrogen levels of women carrying males alter the neurobiological response to estrogen withdrawal following delivery; however, to our knowledge, this has been formally investigated. Additionally, it is notable that the finding of sex differences in maternal hormone levels is not consistent across studies, with some groups detecting no differences by fetal sex [47]. Importantly, we are not aware of research that has examined fetal-sex differences in maternal hormone levels during parturition or the postpartum period, which may be windows of heightened maternal vulnerability based on the findings reported here. Research that repeatedly measures maternal hormone levels across the course of pregnancy and into the postpartum period would provide the means to examine whether fetal sex is associated with altered trajectories of hormone change.

Hormone surges during pregnancy also play a role in maternal immune system shifts that promote tolerance of the fetus [13, 48]. These shifts include an upregulation of generalized inflammatory responses (i.e., release of pro-inflammatory cytokines) towards later pregnancy and into the postpartum period [29]. Depressive disorders are increasingly associated with inflammation [24, 25], providing a putative link between unusually prolonged or excessive immune activation during pregnancy and perinatal depression [15]. Recent research suggests that maternal immune profiles may vary by fetal sex, with women carrying a male fetus having increased levels of pro-inflammatory markers [19, 49]. Sex differences are also evident in studies of the placenta, with male placentas associated with a stronger inflammatory response [18]. Notably, pro-inflammatory cytokines have been shown to increase the activity of serotonin precursors [50], suggesting a role for overlapping biological pathways between the immune and other neurobiological systems. Our finding of a significant association during the postpartum, but not prenatal, period could relate to sex-differential priming of the maternal immune system that only becomes apparent with hormonal fluctuations following delivery. For example, estrogen, which has been shown to vary by fetal sex during pregnancy and withdraws following delivery, binds to receptors expressed on most immune cells [51] and regulates the immunological shift that occurs during pregnancy [29]. While it is plausible that estrogen regulation of immune activity varies by fetal sex, we are not aware of research that has specifically examined interactions between sex, reproductive hormones, immune activity, and PPD susceptibility.

Limited research has also shown fetal-sex differences in maternal levels of a range of other biological molecules, including human chorionic gonadotropin [52], thyroid hormone [53], leptin [54], cortisol [55], and angiogenic factors [19]. In turn, dysfunction of the regulatory systems that orchestrate production and homeostasis of these molecules, including the hypothalamic-pituitary-thyroid and hypothalamic-pituitary-adrenal axes, has been implicated in the pathophysiology of depression [56, 57], including during the postpartum period [16, 58–61]. However, we acknowledge that clinical and mechanistic research examining fetal sex-differential levels of these hormones and other biological effectors in relation to PPD remains limited. Other research has shown sex-differential expression of imprinted genes in the placenta that in turn have been linked to maternal mood disorders [62]. For example, *PEG3* is an imprinted gene that plays important roles in controlling fetal growth and nurturing behaviors. Murine models have demonstrated loss of *Peg3* expression has sexually dimorphic consequences for placental function [63], including the expression of hormones, and influences maternal behavior [64].

In contrast to findings from studies conducted in Western cultures, research conducted in Asia (China, India), Africa (Nigeria), and Turkey has reported female infant sex is positively related to PPD [65–67], with qualitative research supporting a role for male gender preference [68]. For example, a prospective cohort study based in China found an increased odds of PPD among women who gave birth to a female compared to a male infant; however, the association did not hold following adjustment for social support after childbirth [69]. In the United States (U.S.), there is limited research to tie PPD with gender preference; however, we cannot definitively rule out the potential contribution of cultural factors to our findings. For example, econometrics research has identified several differences between U.S. parents of girls versus boys, including a higher divorce rate and differential monetary investment in families [70]. Other research suggests subtle gender preference may vary by nativity, with first- and second-generation American immigrants showing a greater preference for boys [71]. It is thus plausible that cultural factors that track with race/ethnicity or nativity could have contributed to the difference in the magnitude of association that we observed between our diverse cohorts. Moreover, it is well established that in Black and Hispanic communities, boys are often subjected to a range of negative stereotypes and biases related to aggression and violence that place them at greater risk for systemic disadvantages (e.g., school suspensions, incarceration) [72]. It is thus plausible that among minority communities, the increased pressure and parenting stress associated with raising boys may lead to greater psychological stress and precipitate the onset of depressive symptoms. Unfortunately, here we were unable to explore interactions with race/ethnicity or nativity owing to our limited sample size across these categorical variables in combination with sex and levels of prenatal and postnatal depressive symptoms.

Across models, we found that women pregnant with a male had lower, albeit not statistically significant, odds of having depressive symptoms during both the prenatal and postnatal periods, which may indicate a more chronic, severe phenotype of depression. With regard to adverse pregnancy outcomes, male fetuses have been shown to be more vulnerable to a host of prenatal challenges and adverse intra-uterine conditions, including maternal stress [73], nutrient deprivation [74], exposure to environmental toxicants [75], and maternal chronic health conditions [76]. While speculative, it is plausible that this finding reflects a higher incidence of miscarriage among males conceived by women with more severe depression.

### Strengths and limitations

A primary strength of this study relates to the prospective nature and general consistency of results across two distinct pregnancy cohorts, although findings were not significant in ACCESS. We also administered the EPDS at two time points, allowing us to examine the importance of timing and understand differential susceptibility across the prenatal and postnatal periods. Despite this, future studies that include additional screening points across the antenatal, perinatal, and postpartum periods would allow for the effect of timing to be more comprehensively evaluated. Additionally, both cohorts enrolled a high proportion of lower-income, minority women, a demographic with elevated risk for experiencing depression and other mental health disorders [77]. While the diversity of our cohorts is a strength that contributes key research spanning sociodemographic strata, our findings may not generalize to all populations. We also note that Black women enrolled in ACCESS and PRISM were more likely to be excluded from the present analyses due to missing EPDS assessments; this is consistent with other epidemiologic studies that have long cited difficulty retaining African American participants in observational and clinical research [78]. While this selection could plausibly be a threat to the generalizability of our results, we do not expect sex to vary by race/ethnicity, and thus, the internal validity of our findings is unlikely to be compromised. Additionally, while there were no significant differences in EPDS scores between those included and excluded, it is still possible those with the most severe symptoms were lost to follow-up for reasons relating to depression. Other limitations of this study include the use of the EPDS, which is a screening instrument, rather than a diagnostic tool, to identify women with depressive symptoms. However, we do show strong associations between scores on the EPDS and maternal self-reported history of depressive feelings, prior depression diagnosis, and medication use in these samples. Given the stigma associated with perinatal depressions, it is also possible that our use of in-person and telephone interviews to administer the EPDS resulted in underreporting of symptom severity. Additionally, among women who exhibited depressive symptoms during the prenatal period, we do not know whether the onset of symptoms occurred prior to or during pregnancy, which may be an important factor relating to the underlying biological mechanism at play. It is thus possible that misclassification of symptom onset could have biased associations between sex and prenatal depressive symptoms towards the null.

## Conclusion

Expanding literature supports that PPD has a complex and multifactorial etiology that encompasses a range of social, biologic, and psychologic factors. Here, we show that male fetal sex may be one factor contributing to the complex web of determinants underlying individual susceptibility. Current guidelines support tailoring obstetrical care to at-risk groups, such as women of advanced reproductive age (≥ 35 years) [79]. However, whether knowledge of fetal sex should inform follow-up and management practices remains debatable. Current guidelines encourage primary care clinicians to screen all postpartum women for depression, although recommendations about the timing and frequency of screening are unspecific. Our findings support screening women for perinatal depression and suggest that the postnatal period may be a window of heightened vulnerability for women pregnant with a male. Future research is needed to more precisely understand the biological mechanisms underlying fetal sex influences on maternal susceptibility to perinatal depression, including how sex relates to endocrine and immune system changes during the immediate and longer-term postnatal periods. This line of research may identify early biomarkers of heightened risk that may only be elucidated if sex-specific effects are considered.

### Perspectives and significance

This prospective cohort study contributes to an improved understanding of fetal sex as a risk factor for perinatal depression. Specifically, we found that women pregnant with a male fetus had higher odds of developing depression during the postnatal, but not prenatal period. This finding suggests that fetal sex-differential hormonal, immune, or other physiological shifts that occur during late pregnancy, parturition, or the postpartum period may contribute to the onset of postpartum depression and supports the need for future research considering underlying biological mechanisms.

## Supplementary Information

**Additional file 1.** Supplemental Material

## Data Availability

The datasets analyzed during the current study are not publicly available due to confidentiality, but are available from the corresponding author on reasonable request.
