# Supplementary material for: Fetal sex and maternal postpartum depressive symptoms: findings from two prospective pregnancy cohorts
Source: Biol Sex Differ. 2021 Jan 6;12:6. doi: 10.1186/s13293-020-00348-x (PMC7789145; doi:10.1186/s13293-020-00348-x)
Supplement: Supplementary file 1 — Additional file 1. Supplemental Material [file 13293_2020_348_MOESM1_ESM.docx]

**Supplemental Material**

Fetal sex influences on postpartum depressive symptoms: findings from two pregnancy cohorts

Whitney Cowell, Elena Colicino, Talia Askowitz, Farida Nentin, Rosalind J. Wright

Contents

Supplemental Table 1. Characteristics ACCESS mother-child pairs included in analyses versus excluded due to missing prenatal or postnatal EPDS scores.

Supplemental Table 2. Characteristics PRISM mother-child pairs included in analyses versus excluded due to missing prenatal or postnatal EPDS scores.

Supplemental Table 3. Frequency of depressive symptoms by race/ethnicity among women enrolled in the ACCESS and PRISM pregnancy cohorts.

Supplemental Table 4. Odds of maternal depressive symptoms during the prenatal or postnatal periods or both compared to no depressive symptoms during either period in relation feelings of depression, diagnosis of depression, or use of medication for depression.

| Supplemental Table 1. Characteristics of ACCESS mother-child pairs included in analyses versus excluded due to missing prenatal or postnatal EPDS scores. | | | |
| --- | --- | --- | --- |
|  | Excluded (n=624)  N (%)^a^ | Included (n=346)  N (%)^a^ | χ^2^ (*P-value*)^a^ |
| Maternal race/ethnicity |  |  | 7.90 (0.05) |
| White | 54 (9) | 30 (9) |  |
| Black/African American | 200 (34) | 95 (28) |  |
| Hispanic, non-Black | 287 (49) | 200 (59) |  |
| Other | 39 (7) | 16 (5) |  |
| High school or more | 375 (66) | 218 (65) | 0.16 (0.69) |
| Advanced maternal age (>35 years) | 74 (12) | 40 (12) | 0.10 (0.75) |
| Pre-pregnancy overweight/obese (BMI>24) | 380 (73) | 217 (70) | 0.96 (0.33) |
| Smoking during pregnancy | 63 (11) | 41 (12) | 0.27 (0.60) |
| Married/living with partner | 340 (59) | 210 (62) | 0.83 (0.36) |
| Nulliparous | 259 (45) | 146 (43) | 0.36 (0.55) |
| Breastfed > 6 months | 180 (47) | 154 (64) | 16.50 (<0.01) |
| Child sex (male) | 188 (54%) | 315 (50%) | 1.32 (0.25) |
| Prenatal EPDS > 10 | 87 (23) | 91 (26) | 0.82 (0.37) |
| Postnatal EPDS > 10 | 28 (19) | 60 (17) | 0.31 (0.58) |
| Abbreviations: ACCESS, Asthma Coalition on Community Environment and Social; BMI, body mass index; EPDS, Edinburgh Postpartum Depression Scale.  ACCESS (n missing): race/ethnicity (49), education (70), BMI (138), relationship status (6), parity (62), breastfeeding (345), maternal age (26), smoking (71), prenatal EPDS (252), postnatal EPDS (490)  ^a^Chi-square statistic and p-value are for tests examining the difference in characteristic between participants included versus excluded from the analysis. | | | |

| Supplemental Table 2. Characteristics PRISM mother-child pairs included in analyses versus excluded due to missing prenatal or postnatal EPDS scores. | | | |
| --- | --- | --- | --- |
|  | Excluded (n=459)  N (%)^a^ | Included (n=528)  N (%)^a^ | χ^2^ (p-value)^a^ |
| Maternal race/ethnicity |  |  | 18.01 (0.0004) |
| White | 46 (12) | 102 (20) |  |
| Black/African American | 195 (51) | 200 (38) |  |
| Hispanic, non-Black | 124 (33) | 193 (37) |  |
| Other | 15 (4) | 28 (5) |  |
| High school or more | 311 (82) | 400 (77) | 3.12 (0.08) |
| Advanced maternal age (>35 years) | 67 (15) | 103 (20) | 4.09 (0.04) |
| Pre-pregnancy overweight/obese (BMI>24) | 206 (54) | 278 (53) | 0.13 (0.72) |
| Smoking during pregnancy | 37 (9.54) | 64 (12) | 1.52 (0.22) |
| Married/living with partner | 250 (66) | 362 (69) | 1.40 (0.24) |
| Nulliparous | 139 (33) | 160 (30) | 0.54 (0.46) |
| Breastfed > 6 months | 77 (26) | 293 (57) | 69.62 (<0.0001) |
| Child sex (male) | 234 (51) | 273 (52) | 0.05 (0.82) |
| Prenatal EPDS > 10 | 69 (24) | 114 (22) | 0.64 (0.42) |
| Postnatal EPDS > 10 | 14 (11) | 67 (13) | 0.21 (0.65) |
| Abbreviations: BMI, body mass index; EPDS, Edinburgh Postpartum Depression Scale; PRISM, PRogramming of Intergenerational Stress Mechanisms.  PRISM (n missing): race/ethnicity (84), education (90), BMI (85), relationship status (6), parity (37), breastfeeding (177), maternal age (1), smoking (71), prenatal EPDS (172), postnatal EPDS (334).  ^a^Chi-square statistic and p-value are for tests examining the difference in characteristic between participants included versus excluded from the analysis. | | | |

| Supplemental Table 3. Frequency of depressive symptoms, defined as an EPDS score > 10, by race/ethnicity among women enrolled in the ACCESS and PRISM pregnancy cohorts. | | | | |
| --- | --- | --- | --- | --- |
|  | ACCESS | | PRISM | |
|  | Prenatal | Postnatal | Prenatal | Postnatal |
| White, non-Hispanic | 8 (27%) | 7 (23%) | 12 (12%) | 8 (8%) |
| Black/Black-Hispanic | 32 (34%) | 27 (28%) | 51 (26%) | 23 (12%) |
| Hispanic, non-Black | 44 (22%) | 23 (12%) | 43 (22%) | 34 (18%) |
| Other | 7 (44%) | 3 (19%) | 7 (25%) | 2 (7%) |
| χ^2^ (p-value) | 7.00 (0.072) | 13.50 (0.004) | 7.87 (0.049) | 7.36 (0.061) |
| Abbreviations: ACCESS, Asthma Coalition on Community Environment and Social; EPDS, Edinburgh Postpartum Depression Scale; PRISM, PRogramming of Intergenerational Stress Mechanisms. | | | | |

| Supplemental Table 4. Odds [odds ratio (95% confidence interval) of maternal depressive symptoms^a^ during the prenatal or postnatal periods or both compared to no depressive symptoms during either period in relation feelings of depression, diagnosis of depression, or use of medication for depression in the ACCESS and PRISM pregnancy cohorts. | | | |
| --- | --- | --- | --- |
|  | Feel depressed before pregnancy^c^ | Depression diagnosis^d^ | Depression medication^e^ |
| ACCESS |  |  |  |
| No symptoms (n=228, 66%) | 1.00 (Reference) | 1.00 (Reference) | N/A |
| Prenatal only (n=58, 17%) | 3.96 (2.17, 7.23) | 2.32 (1.20, 4.49) | N/A |
| Postnatal only (n=27, 8%) | 2.08 (0.92, 4.69) | 1.17 (0.42, 3.30) | N/A |
| Prenatal and postnatal (n=33, 9%) | 5.73 (2.57, 12.77) | 5.49 (2.55, 11.83) | N/A |
| PRISM |  |  |  |
| No symptoms (n=390, 74%) | 1.00 (Reference) | 1.00 (Reference) | 1.00 (Reference) |
| Prenatal only (n=71, 13%) | 3.30 (1.96, 5.55) | 3.01 (1.74, 5.21) | 0.82 (0.24, 2.82) |
| Postnatal only (n=24, 5%) | 5.53 (2.24, 13.69) | 2.95 (1.24, 7.02) | 3.70 (1.16, 11.85) |
| Prenatal and postnatal (n=43, 8%) | 14.04 (5.78, 34.17) | 7.51 (3.86, 14.62) | 4.23 (1.74, 10.30) |
| Abbreviations: ACCESS, Asthma Coalition on Community Environment and Social; PRISM, PRogramming of Intergenerational Stress Mechanisms.  ^a^Defined as an Edinburgh Postpartum Depression Scale score >10.  ^b^Adjusted for: race/ethnicity, age, education, parity, relationship status, smoking during pregnancy, body mass index, and breastfeeding duration.  ^c^Assessed with the question: “Before this pregnancy, was there ever a period of time when you were feeling depressed or down or when you lost interest in pleasurable activities most of the day, nearly every day, for at least 2 weeks?”,  ^d^Assessed with the question: “Before this pregnancy, did you ever see a health care professional who said that you were depressed?”  ^e^Assessed with the question: “During your pregnancy, did you take prescription medication for depression or to help you sleep?”. Question asked in PRISM cohort only. | | | |
